# STAT, Wingless, and Nurf-38 determine the accuracy of regeneration after radiation damage in *Drosophila*

**DOI:** 10.1371/journal.pgen.1007055

**Published:** 2017-10-13

**Authors:** Shilpi Verghese, Tin Tin Su

**Affiliations:** 1 Department of Molecular, Cellular and Developmental Biology, UCB, University of Colorado, Boulder, CO, United States of America; 2 University of Colorado Comprehensive Cancer Center, Anschutz Medical Campus, Aurora, CO, United States of America; Geisel School of Medicine at Dartmouth, UNITED STATES

## Abstract

We report here a study of regeneration in *Drosophila* larval wing imaginal discs after damage by ionizing radiation. We detected faithful regeneration that restored a wing disc and abnormal regeneration that produced an extra wing disc. We describe a sequence of changes in cell number, location and fate that occur to produce an ectopic disc. We identified a group of cells that not only participate in ectopic disc formation but also recruit others to do so. STAT92E (*Drosophila* STAT3/5) and Nurf-38, which encodes a member of the Nucleosome Remodeling Factor complex, oppose each other in these cells to modulate the frequency of ectopic disc growth. The picture that emerges is one in which activities like STAT increase after radiation damage and fulfill essential roles in rebuilding the tissue. But such activities must be kept in check so that one and only one wing disc is regenerated.

## Author summary

Accuracy in regeneration ensures that the original structures are restored, no more and no less. Prior studies in the wing primordia of *Drosophila melanogaster* larvae that have been damaged by high energy radiation show that regeneration occurs to restore the original structure. We report here that, in the same experimental system, abnormal regeneration can also occur to produce extra wing structures. We describe a series of cell rearrangements and fate changes that underlie abnormal regeneration, and identify genes responsible for these events. Modulation of such genes have the potential to mitigate abnormal regeneration that occurs after radiation damage to produce such side effects as ulcers and fibrosis.

## Introduction

Ionizing radiation (IR) is one of three main modalities in the treatment of cancer, the others being surgery and chemotherapy. Therapeutic effect of IR relies on its ability to kill cells. But what remains could regenerate a tumor, leading to treatment failure. In parallel, incorrect healing of normal tissues after collateral damage by radiation therapy contributes to side effects such as fibrosis and ulcers [for example, [1, 2]]. Understanding how tumors and normal tissues regenerate after damage by IR could help us make radiation therapy of tumors more effective while reducing unwanted side effects. This study aims to use a powerful genetic model, Drosophila, to identify and characterize genes needed for faithful regeneration after radiation damage.

Imaginal discs of *Drosophila melanogaster* larvae are precursors of adult organs. Because of their high regenerative ability, imaginal discs have been used in studies of tissue regeneration. In classical and recent studies, imaginal discs were surgically fragmented and cultured in adult or larval hosts (e.g. [3]; reviewed in [4, 5]). Faithful regeneration restored the original structure but abnormal regeneration that produced duplications, transdeterminations and multiplications were also recorded. In leg discs, for example, medial (anterior) fragments typically regenerated whole leg discs whereas lateral (posterior) fragments typically duplicated the surviving structure [3]. Transdetermination in which the regenerated parts assume the identity of another disc was also frequent in the classical studies of surgically ablated leg discs (taking on wing fate). Multiplication produced too many copies of a normal part in the regenerated structure, for example sensilla trichodea (sense organs) in the leg [3].

Another model of regeneration uses genetic means to kill cells in a specific compartment, for example by temporally regulated expression in the wing pouch of pro-apoptotic genes such as *eiger*, *hid or rpr* [6, 7]. The discs with ablated parts reside in situ and regeneration is monitored after shutting off the death-inducing gene. Genetic and molecular analysis identified Wg (*Drosophila* Wnt-1) and STAT92E as key players in regeneration in both surgically and genetically ablated discs (e.g. [5, 7–11]). STAT92E (to be called STAT here) is the sole *Drosophila* STAT and is an orthologue of mammalian STAT3/5. Wg and STAT, for example, were found to be activated by JNK activity at the wound site and promoted cell proliferation and blastema formation that underlies regeneration after surgical ablation [8].

We have been studying regeneration of imaginal discs after damage by ionizing radiation (IR). Unlike surgical or genetic ablation, IR induces apoptosis that is scattered throughout the disc. Larvae in which about half of the cells in each imaginal discs have been killed by IR (typically 4000R of X-rays) regenerate to produce a viable fertile adult fly [12, 13]. While IR-induced apoptosis is scattered, the distribution is not random. We found, for example, that in wing imaginal discs, cells of the future hinge, particularly those in the dorsal half, are more resistant to IR-induced apoptosis than cells of the wing pouch [14]. The hinge cells then participate in the regeneration of the wing pouch that suffers more IR-induced apoptosis than the hinge. The hinge also displays other characteristics such as different apical-basal organization of cells and the presence of ‘tumor hot-spots’ that are particularly prone to neoplastic transformation [15]. We identified two functions of Wg and STAT in the hinge. First, Wg and STAT protect hinge cells from IR-induced apoptosis. Second, Wg and STAT are needed to promote the translocation of these cells towards the pouch, thereby facilitating regeneration of the wing disc after radiation damage.

Here, we report that regeneration after IR damage can occur faithfully to restore the wing disc or produce abnormal structures, just like abnormal regeneration after surgical ablation produces duplications, transdeterminations and multiplications. We studied regeneration of irradiated larval wing discs in a time course using a lineage tracing system, and identified a sequence of cellular events that produced a second, ectopic wing disc. We used tissue-specific expression of RNAi to identify cell-autonomous roles for epigenetic regulators, STAT, and Wg in this process. We found that faithful regeneration (to restore the primary wing disc) and abnormal regeneration (to produce an ectopic wing disc) have common as well as distinct genetic requirements. All genetic components we identified are conserved in mammals, making it possible to test their roles in normal and abnormal regeneration after radiation damage in those systems in future studies.

## Results

### Irradiation produces extra growth during regeneration

**Fig 1:**
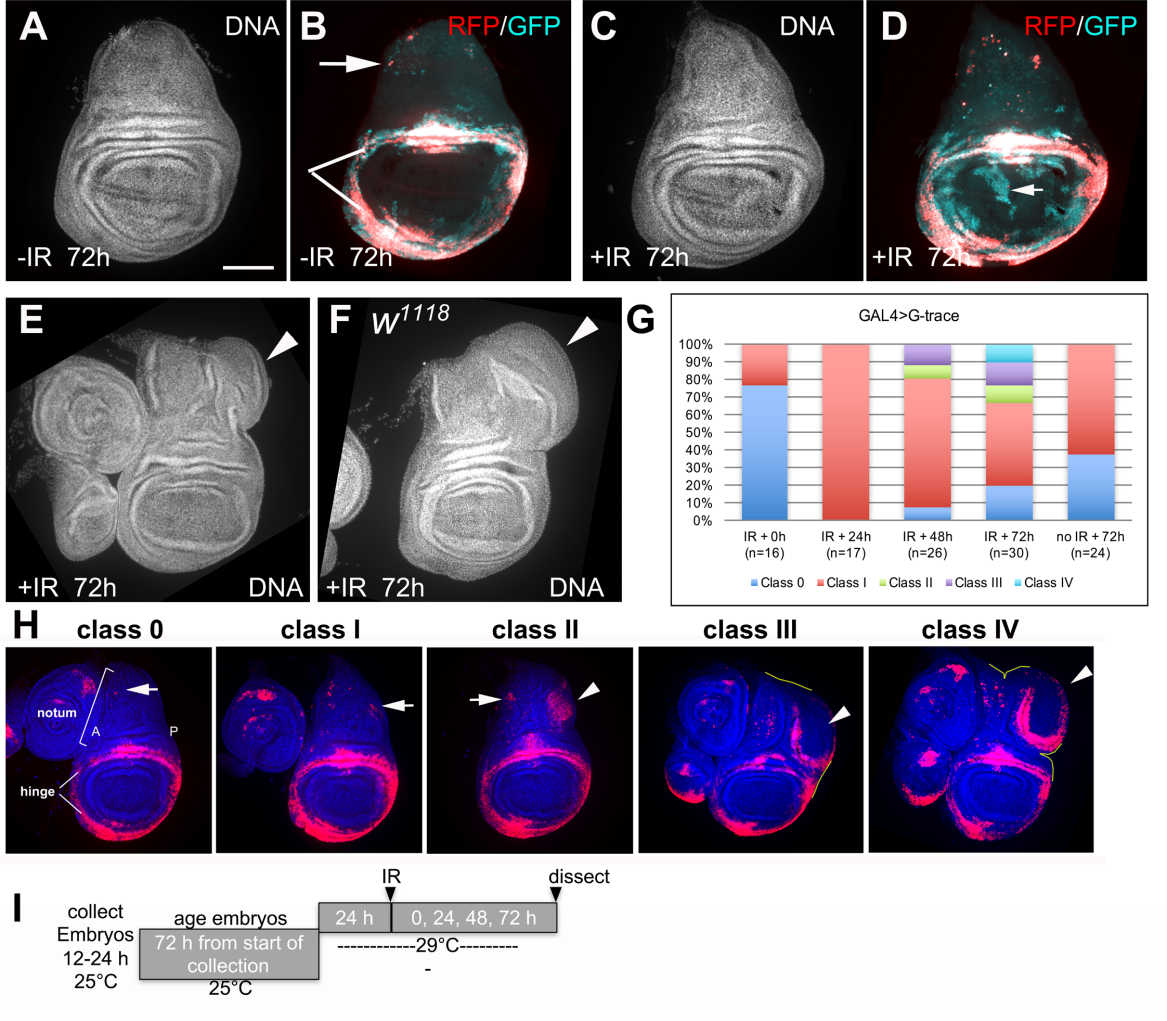
Radiation induces extra growth in the wing disc. 72–96 h old larvae treated with the temperature shift protocol as shown in panel I were irradiated with 4000R of X-rays. The discs were dissected 72 h after irradiation, fixed and stained for DNA and visualized for RFP/GFP when present. All discs shown were of the genotype 30A-GAL4>UAS-RFP, G-trace/+; GAL80^ts^/+except (F) that shows a *w*^*1118*^ disc. Scale bar = 100 μm in (A-F); 120 μm in (H). (A-D) Discs at 72 h after irradiation with 0 (-IR) or 4000R of X-rays (+IR). Regenerated discs appear normal by DNA stain (C) but the pouches were rebuilt from hinge cells that translocated and changed fate (arrow in D). (E) A regenerated disc with ectopic growth (arrowhead). (F) A regenerated *w*^*1118*^ disc with ectopic growth (arrowhead). (G) The frequency of disc classes at various times after irradiation in 30A-GAL4>G-trace discs. n = number of discs examined. 0 h = immediately before irradiation. Data from one time course with a single cohort of larvae is shown, but similar data were seen in two other time courses (biological replicates). (H) Representative images illustrate Classes 0-IV. (I) The temperature shift protocol.

In our studies of wing disc regeneration after radiation damage, we used the 30A-GAL4 driver, in conjunction with GAL80^ts^, to direct gene expression to the hinge, which is the site of IR-resistant cells that participate in regeneration [14]. 4000R of X-rays were used in all experiments in this work. We expressed a published G-trace system in which UAS-RFP serves as a real-time marker (Fig 1B and 1H), while clonally stable GFP expression is induced by UAS-FRT mediated recombination [16]. When used in conjunction with a tissue/cell type-specific GAL4 driver such as 30A-GAL4, cells in which GAL4 is active express RFP. 30A-GAL4 is active in parts of the hinge (indicated with white lines in Fig 1B, see also Fig 1H) and a few cells of the notum (arrow in Fig 1B, see also Fig 1 H). These cells also undergo FRT-mediated recombination to stably express GFP from a Ubiquitin promoter. If these cells or their descendants change fate and lose GAL4 activity, RFP would cease to express but GFP would still be expressed, allowing us to follow cells through fate changes. GAL4 activity is further controlled temporally by repressing it with a temperature sensitive GAL80. Only upon a shift to 29°C to inactivate GAL80^ts^ (Fig 1I), would GAL4 become active to lineage mark cells of interest. The larvae were then irradiated and the fate of labelled cells followed in a time course. In these studies, regeneration restores normal appearance by DNA stain (Fig 1, compare 1A to 1C), but regenerated wing pouches were composed in part of cells derived from the hinge but have lost their hinge fate (RFP^-^GFP^+^; arrow in Fig 1D) [14]. Without irradiation, cell fates are relatively more stable (Fig 1B).

In this experimental system, we noted that some of the wing discs in irradiated samples had ectopic growths (Fig 1E, arrowhead, quantified in G and classified in H). Wing discs from similarly treated wild type *w*^*1118*^ larvae also produced ectopic growths (Fig 1F, arrowhead). IR-induced ectopic growths were dependent on the temperature shift protocol (Fig 1I). This protocol allowed larval growth and disc development before shifting to 29°C to inactivate GAL80^ts^ and activate GAL4. Ectopic growths did not form if the larvae were kept at 25°C throughout the experiment; 45/45 discs were class 0/I in two separate experiments. While we do not know why the production of ectopic discs depended on a temperature shift, the ability to produce them provides us with an opportunity to study this mode of regeneration. It should be possible to test the role of temperature-induced molecular changes such as the heat shock response in the future.

We used 30A-GAL4>RFP expression pattern to classify discs with ectopic growths. RFP was predominantly in the hinge but was also in a few scattered cells in the notum (Fig 1H). All classes of discs showed similar RFP signal in the hinge but differed in the number and organization of RFP^+^ cells in the notum. Class 0 discs showed <10 RFP^+^ cells (arrow), almost all in the anterior (A) half of the notum. Class I discs showed more RFP^+^ cells that have spread into the posterior (P) half of the notum (arrows). Class II discs showed a cluster of RFP^+^ cells near the dorsal-posterior edge of the notum (arrowhead) in addition to scattered RFP^+^ cells in the notum (arrow). Class III discs showed RFP^+^ cells in a ring that is often incomplete (arrowhead), also near the dorsal posterior edge of the notum, that still shares a continuous boundary with the rest of the disc (the disc boundary traced with a yellow line appears continuous). Class IV discs showed similar rings of RFP^+^ cells (arrowhead) that have grown into a structure no longer confined within the notum (the disc boundary traced with a yellow line is dis-continuous). Quantitative analysis showed that all un-irradiated discs were class 0 or I; classes II-IV are strictly IR-dependent (Fig 1G, compare 72 h -/+IR, the last two bars).

To understand how different classes of ectopic growth relate to each other, we followed 30A-GAL4>G-trace discs in a time course after irradiation. Immediately before irradiation (0 h), the discs were predominantly class 0 (Fig 1G, IR+ 0h). All discs were class I at 24 h after IR. Classes II and III appeared at 48 h after IR, followed by classes IV at 72 h after IR. All irradiated larvae entered the pupal stage subsequently. The complete absence of class 0 discs at 24 h after irradiation is striking because by 72 h after irradiation, all five classes were represented (Fig 1G). At the intermediate time point of 48 h, we saw classes II and III but not yet IV. We interpret these data to mean that between 24 and 72 h after irradiation, some class I discs regressed to class 0 while others progressed to classes II and III and, subsequently, class IV.

The frequency of class III and IV discs at 72 h after irradiation in 30A-GAL4>G-trace discs was 20.9% (Fig 1G). *w*^*1118*^ discs lacked RFP and could not be classified as in Fig 1H, but the frequency of discs with obvious overgrowths as in Fig 1E and 1F was similar; 38/183 or 20.8% in four independent experiments using the protocol in Fig 1I showed obvious ectopic growths, while none did in un-irradiated samples (0/153). Similar frequency of ectopic discs in 30A-GAL4>G-trace and *w*^*1118*^ discs means that ectopic growths are not due to 30A-GAL4>G-trace transgenes.

### Ectopic growths are wing discs derived in part from RFP^+^ cells of the notum

**Fig 2:**
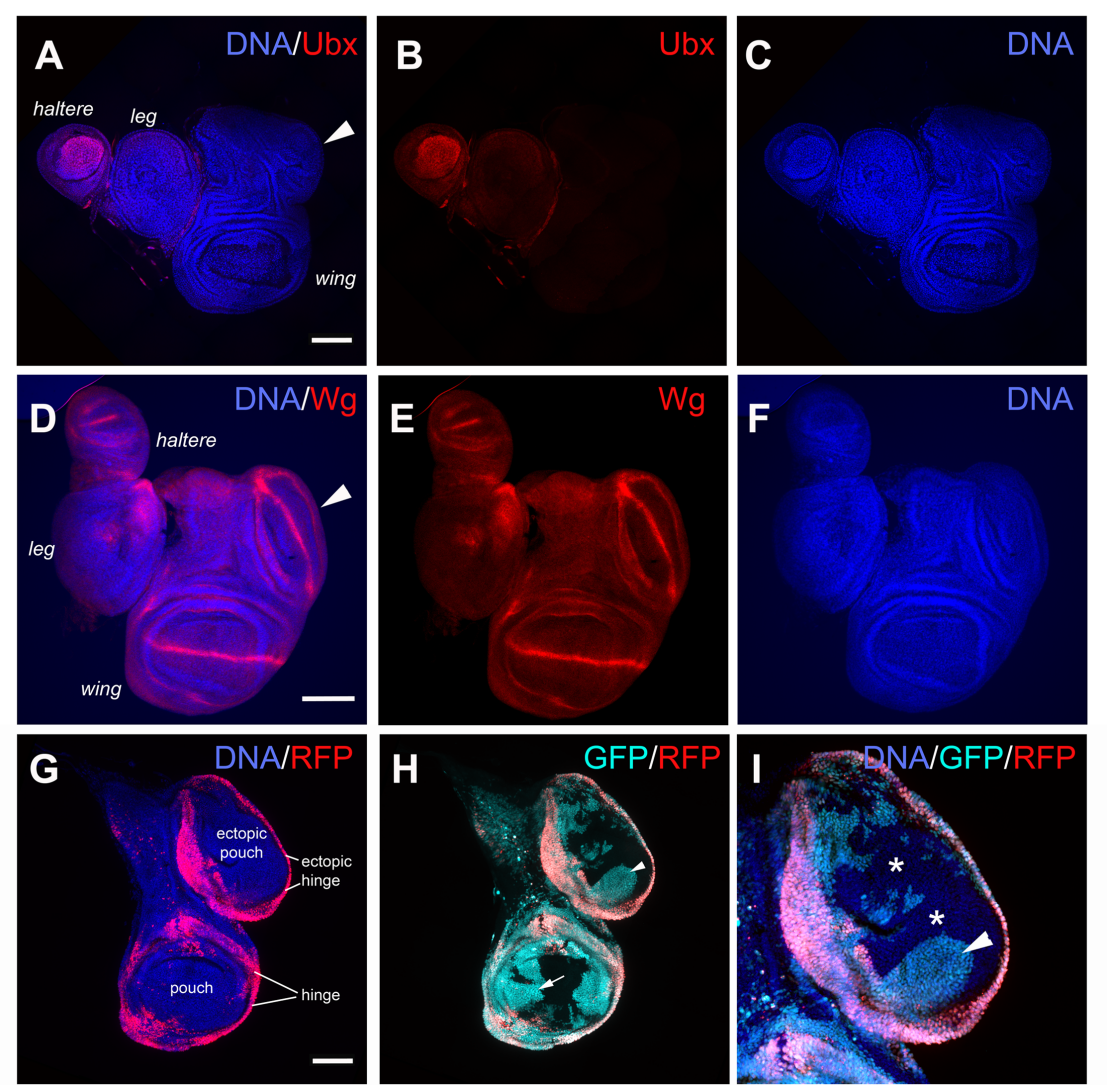
The extra growths in regenerated discs are ectopic wing discs that originate from RFP^+^ cells in the notum. Larvae were treated as shown in Fig 1I. The discs were dissected 72 h after irradiation, fixed and stained for DNA and visualized for RFP. The discs were also stained with antibodies against Ubx and Wg as indicated. All are from 30A-GAL4>UAS-RFP, G-trace/+; GAL80^ts^/+ larvae. The discs are shown Anterior left and Dorsal up. Scale bar = 100 μm in all panels except in (I) where it is 50 μm. (A-C) Ubx signal is present in the haltere disc but not in the ectopic disc. (D-F) Wg pattern in the ectopic disc is unlike in the leg disc. (G-I) An example of a class IV disc in which cells of the ectopic disc (magnified 2X in I) are composed of RFP^+^ cells in the ectopic hinge (G), RFP^-^GFP^+^ cells (arrowheads in H and I) and RFP^-^GFP^-^ cells (* in I).

IR-induced ectopic growths lacked Ubx and were therefore not haltere discs (Fig 2A–2C, arrowhead) [17]. The expression of Wg protein in a theta (θ) pattern in the ectopic growths (Fig 2D–2F, arrowhead) matched that of wing and haltere discs but not leg discs where it is a wedge. Wg pattern and the absence of Ubx indicated that ectopic growths were ectopic wing discs. All ectopic wing discs were found at the dorsal-posterior edge of the notum (n>300 discs examined), at the same location as RFP^+^ cell clusters in class II discs (Fig 1H, arrowhead in ‘class II’). We hypothesized that such cell clusters matured into ectopic discs to produce class III and IV discs. To test the idea, we followed the fate of RFP^+^ cells using the GFP lineage tracer [16] (Fig 2H and 2I). We found that ectopic pouches included descendants of cells that used to express RFP (RFP^-^GFP^+^, arrowhead in Fig 2H). This is similar to what we saw in the regenerated wing pouch after irradiation, that it was composed of GFP^+^RFP^-^ former hinge cells that lost their hinge fate [14] (arrow in Fig 1D). The key difference was that fate change and pouch-building occurred days after irradiation and in an ectopic wing disc that did not suffer IR-induced damage. Furthermore, ectopic wing discs also included RFP^-^GFP^-^ cells (* in Fig 2I). These never expressed RFP (because they lacked the GFP lineage marker) and therefore must have originated either from RFP^-^ cells of the primary notum or the primary pouch. We had used the G-trace system previously to test if pouch cells relocated after irradiation; they did not [14]. Therefore, the notum was the likely origin of RFP^-^GFP^-^ cells in the ectopic disc. These findings are consistent with the idea that ectopic wing discs originated from a combination of RFP^+^ cell cluster near the dorsal posterior margin of the notum in class II discs and un-related (RFP^-^GFP^-^) notum cells.

### Initial changes in the notum accompanies cell number increase through mitosis

**Fig 3:**
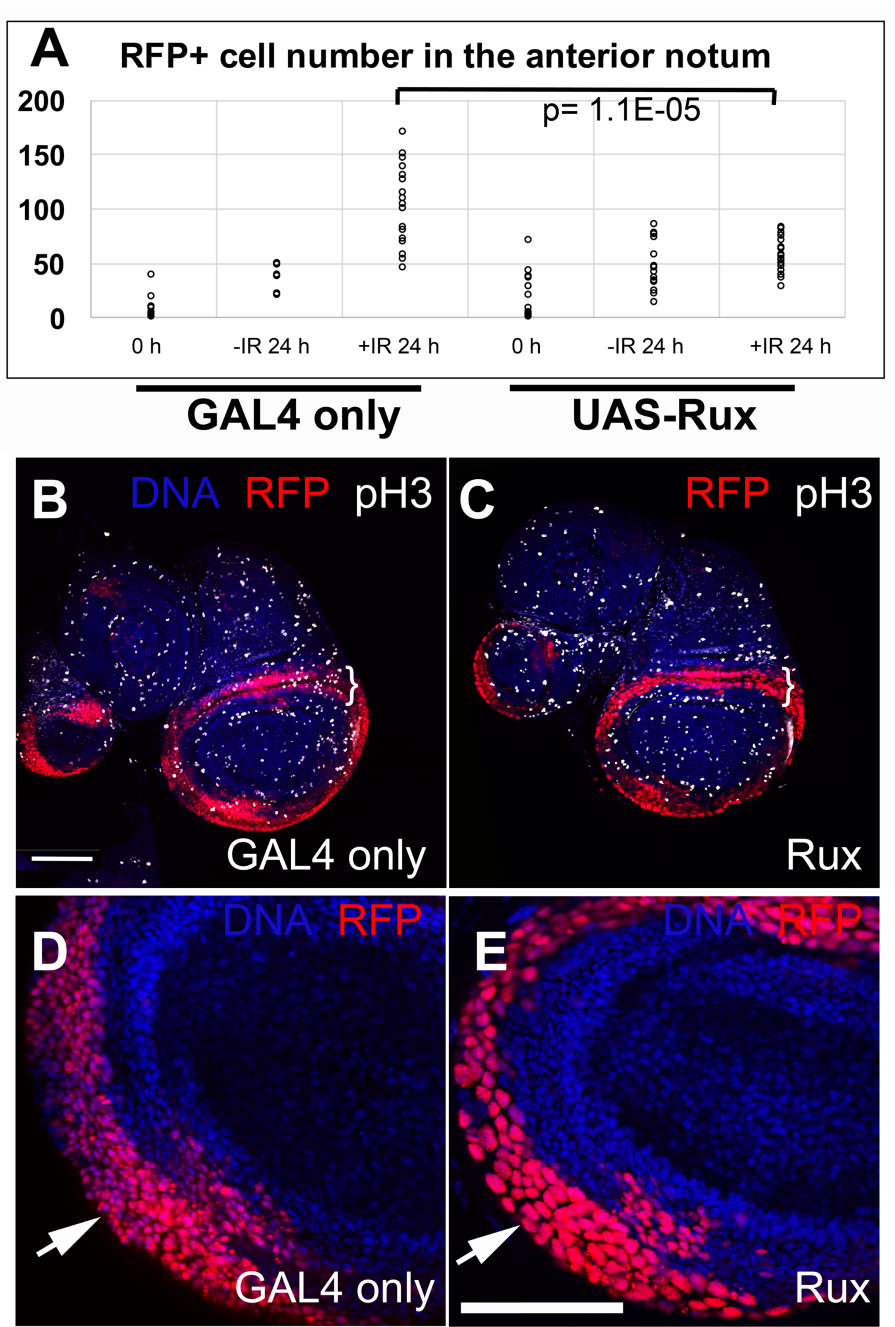
Rux blocked mitosis and stabilized cell number. Larvae were treated as shown in Fig 1I. The discs were fixed and stained for DNA and with an antibody against phospho-S10-Histone H3 (pH3), a marker of mitosis, and visualized also for RFP. Genotype: 30A-GAL4>UAS-RFP, G-trace/+; GAL80^ts^/+ (GAL4 only) or 30A-GAL4>UAS-RFP, G-trace/UAS-Rux; GAL80^ts^/+ (Rux). Scale bar = 50 μm. (A) Quantification of RFP^+^ cell number in the anterior half of the notum at the time of irradiation (0 h) or 24 h later. The number of discs analyzed in two-four different experiments (biological replicates) were: for GAL4 only T0 (24), T24-IR (6), T24+IR (19); Rux T0 (14), T24-IR (15), T24+IR (18). Student t-test was used for statistical significance. Sample notums used for quantification are shown in S1 Fig. (B-E) Wing discs at 0 h (immediately before irradiation). pH3^+^ cells are absent in the 30A-GAL4 domain marked with RFP in (C). A single confocal slice is shown. (D-E) Nuclear RFP shows that cells in the 30A-GAL4 domain have large nuclei compared to those in the GAL4 only controls (arrows). DNA signal shows similar-sized nuclei outside the 30A-GAL4 domain in both discs.

The abundance of class 0 discs at the time of irradiation and the complete incidence of class I discs at 24 h after irradiation (Fig 1G) reflects the fact that RFP^+^ cells in the notum increased in number during this period. Quantification of RFP^+^ cell number confirmed this (Fig 3A, compare -/+IR 24 h in GAL4 only samples, p = 1.05E-05 in 2-tailed t-test). Sample images used in the quantification are shown in S1 Fig. To ask if this increase was due to mitosis, we co-expressed Rux in RFP^+^ cells. Rux is an inhibitor of cyclin/cdk complexes, with particular activity towards cyclin A/cdk1, which plays a mitotic role in *Drosophila* [18, 19]. Rux is expected to block mitosis without affecting S phase. We confirmed this by staining for phospho-S10-Histone H3, a mitotic marker; we saw reduced mitoses in the 30A domain (Fig 3B and 3C, brackets) and cells with large nuclei that resulted from repeated S phases without mitosis (Fig 3, arrows compare red nuclei in D and E). Co-expression of Rux abolished the IR-induced increase in RFP^+^ cells in the notum (Fig 3A, compare UAS-Rux -IR 24h to +IR 24 h, p = 0.16 by 2-tailed t-test), suggesting that cell number increase within 24 h after irradiation was due to increased mitoses. Increased proliferation after IR-induced cell death would be similar to the phenomenon of ‘accelerated proliferation’ in which surviving cancer cells proliferate faster than before irradiation (pg. 384 in [20]). Mitotic index also increased in larval imaginal discs recovering from irradiation, with the increase occurring throughout the wing disc [12, 13]. Cell death by other means can also result in increased proliferation in *Drosophila* imaginal discs, as in the phenomenon of compensatory and apoptosis-induced proliferation (e.g. [5, 21–23] for review).

### Ectopic wing discs can form while mitosis is prevented with Rux

**Fig 4:**
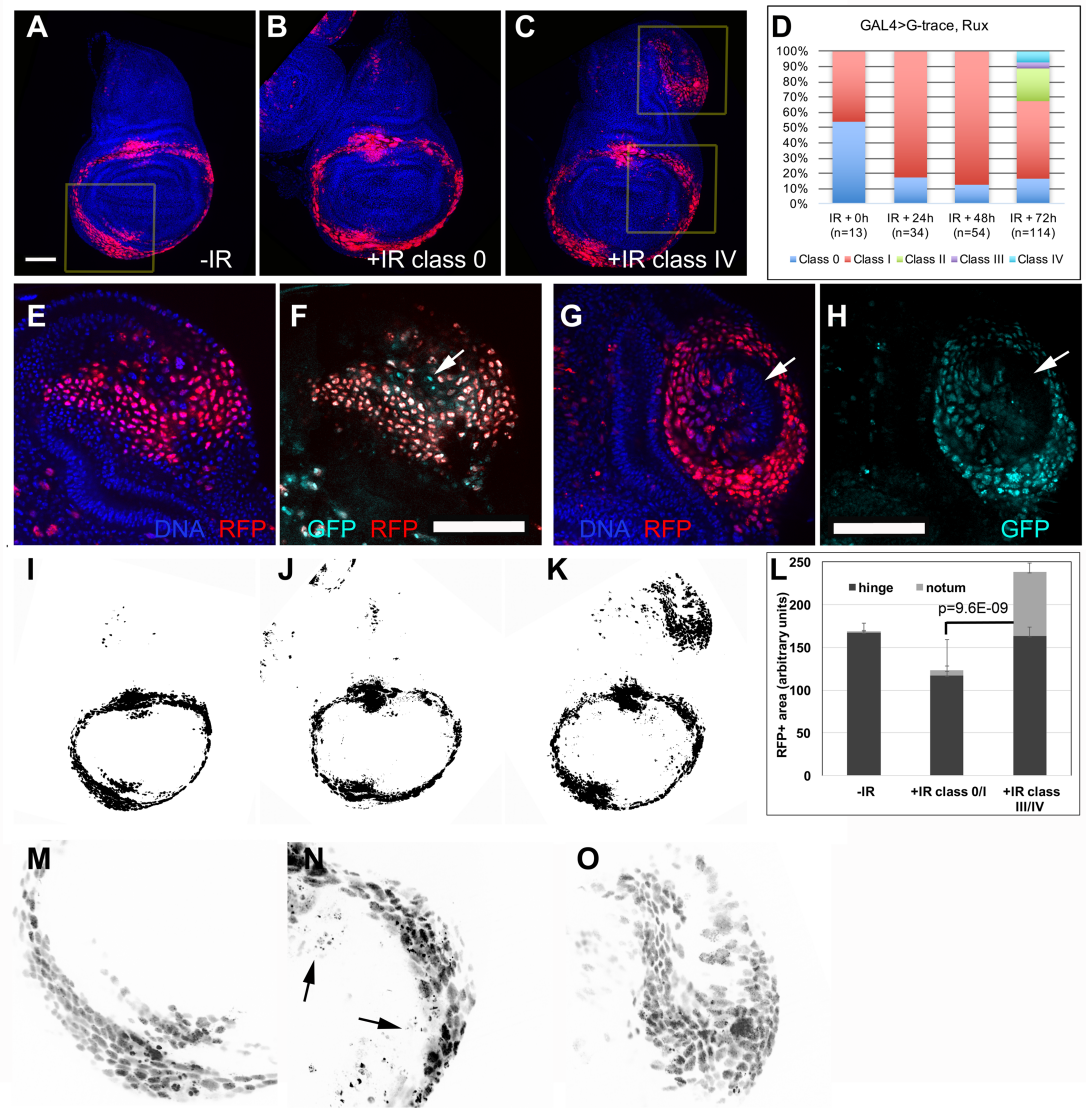
Ectopic discs formed even when mitosis was inhibited. Larvae of the genotype 30A-GAL4>UAS-RFP, G-trace/UAS-Rux; GAL80^ts^/+ were treated as shown in Fig 1I and dissected at various times after irradiation. The discs were fixed and stained for DNA and visualized for RFP and GFP. A-C show z-projected images. E-H show a single z-section from two different discs, for clarity. Scale bar in A (50 μm) applies to panels A-C and I-K; scale bar in F (also 50 μm) applies to panels E-H and M-O. (A) A wing disc at 0 h (immediately before irradiation). RFP signal in the boxed area is shown magnified in M. (B) A class 0 disc at 72 h after irradiation. (C) A class IV disc at 72 h after irradiation. RFP signal in the boxed areas is shown magnified in N-O. (D) Quantification of different classes from two biological replicates combined. (E-H) Ectopic discs formed under Rux expression also contained RFP^+^GFP^+^ cells (most RFP^+^ cells in E and F), RFP^-^GFP^+^ cells (arrow in F) and RFP^-^GFP^-^ cells (arrows in G-H). (I-K) Images in A-C were threshold-set in Image J. (L) Area above the threshold from images such as those in (I-K) were quantified in Image J and averaged. n = 6 per group in two different experiments. Error bar = 1 STD. The p value represents the comparison of the notum areas in–IR against +IR class III/IV. The p value for the hinge in the same pair-wise comparison was 0.74. (M-O) Boxed areas in A (M) or C (N-O) are shown magnified and black/white inverted for clarity. Arrows in N point to RFP dots that we infer represent fragmented nuclei.

Even with Rux-induced inhibition of mitosis, classes II-IV discs were produced, although with a delay and with reduced frequency (Fig 4A–4C, compare 4D to Fig 1G). Lineage tracing experiments showed that ectopic discs formed under Rux expression were also composed of RFP^+^GFP^+^, RFP^-^GFP^+^ and RFP^-^GFP^-^ cells, just like the ectopic discs of GAL4 only control larvae (Fig 4E–4H). Furthermore, ectopic disc formation accompanied a large increase in RFP^+^ cell number in the notum of 30A>Rux discs between 24 and 72 h after irradiation; we counted 281±61 nuclear RFP^+^ cells in the ectopic growths of class II-IV 30A>Rux discs at IR+72 h (n = 6 in three biological replicates). In contrast, RFP^+^ cells in the notum in 30A>Rux discs numbered only 57±16 at IR+24 h (Fig 3A). This increase occurred without apparent changes in the rest of the disc. For example, Fig 4A–4C were threshold-adjusted and shown in Fig 4I–4K. Where did these extra ~200 RFP^+^ cells come from in the span of 48 h while mitosis was prevented by Rux? We do not favor the possibility that they are migrants from the primary hinge, for the following reasons. At 48–72 h after irradiation, un-irradiated hinges showed 443±111 cells (n = 10 in two different biological replicate experiments; Fig 4M shows an example of an optical slice used for nuclear counting). To produce ~200 cells in the notum, about half must have translocated. But in all our time courses (Fig 1G), we saw no evidence of hinge cells translocating into the notum/ectopic pouch even though the translocation in the other direction was readily detected (Fig 1D, arrow). We also attempted to count RFP^+^ cells in the primary hinge, to detect any depletion. But unlike in the ectopic hinge where RFP^+^ nuclei were discrete (e.g. Fig 4O), RFP^+^ nuclei in the primary hinge of irradiated discs were often fragmented, perhaps due to IR-induced cell death (arrows in Fig 4N). This prevented us from counting nuclei in the primary hinge with confidence. Instead, we quantified the RFP^+^ area in the hinge from threshold-adjusted images such as those in Fig 4I–4K. While a significant increase in the notum was detected (Fig 4L), there was no evidence of depletion of RFP^+^ cells in the hinge of class III/IV discs (compare–IR to +IR class III/IV). We also saw no signs of hypertrophy in irradiated hinges of class III/IV discs (compare nuclei size in Fig 4M and 4N), ruling out the possibility that while half the hinge cells translocated to the notum, the remainder underwent extra S phases to fill in the gap. Taken together, these results do not favor the possibility that RFP^+^ cells from the primary hinge translocated into the notum to produce ectopic discs. This leaves de novo induction of the RFP^+^ fate in notum cells as a likely possibility, as discussed in DISCUSSION.

### STAT is required cell-autonomously for ectopic growth while Wg also makes a contribution

**Fig 5:**
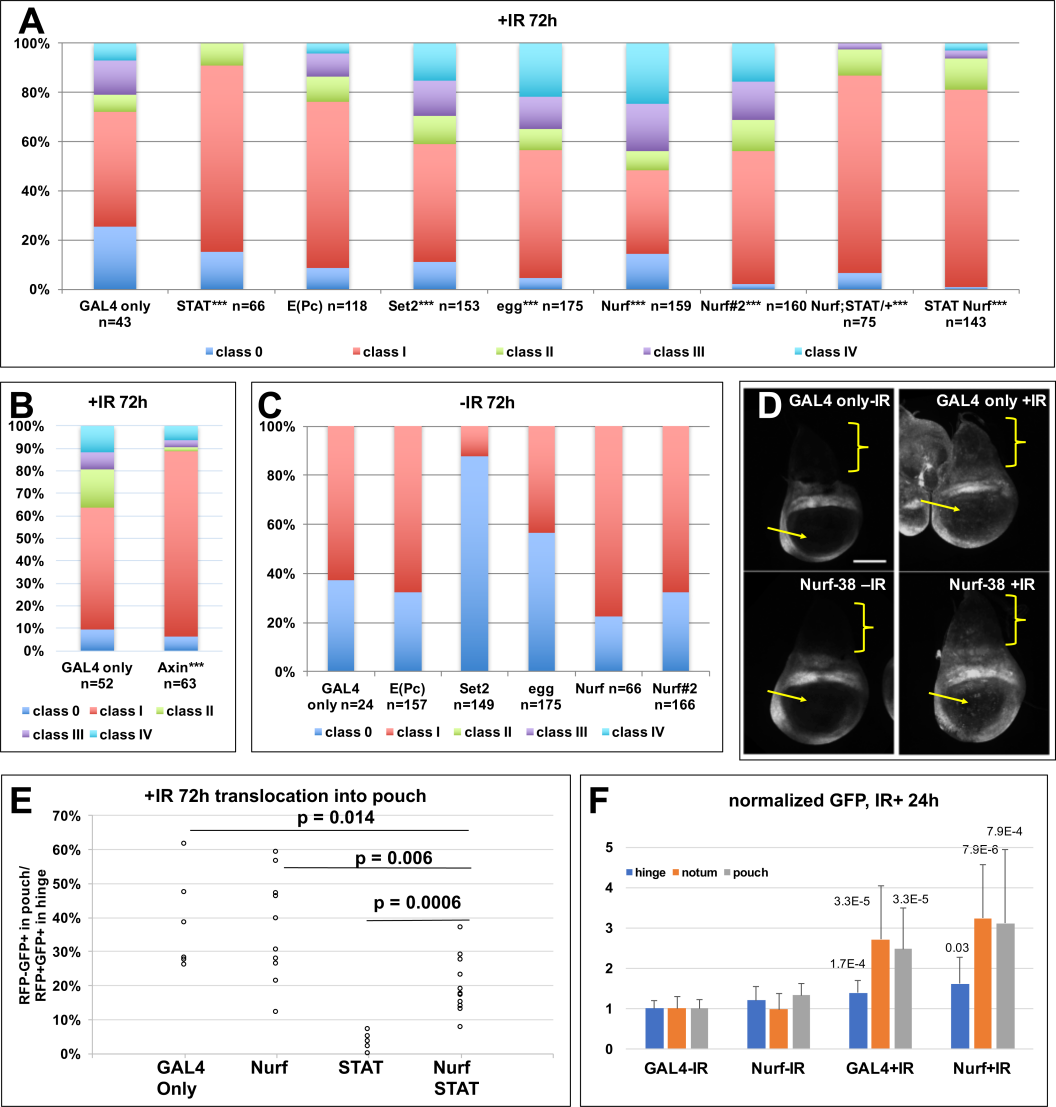
The frequency of ectopic discs is modulated by Wg, STAT and epigenetic regulators. Larvae were treated as in Fig 1I except for a modification in Axin experiments (see text). The discs were dissected 72 h (A-C) or 24 h (D) after irradiation, fixed and stained for DNA and visualized for RFP/GFP when present. **p<0.01, ***p<0.001 vs. GAL4 only +IR (see S1 Table for Chi-square analysis and p values for different pair-wise comparisons). (A-C) The frequency of disc classes from 2–4 independent experiments (biological replicates). Genotypes were: GAL4 only = 30A-GAL4>UAS-RFP, G-trace/+; GAL80^ts^/+ / STAT = UAS-STAT RNAi/+; 30A-GAL4>UAS-RFP, G-trace/+; GAL80^ts^/+ / E(Pc) = 30A-GAL4>UAS-RFP, G-trace/+; GAL80^ts^/UAS-E(Pc) RNAi / Set2 = 30A-GAL4>UAS-RFP, G-trace/ UAS-Set2 RNAi; GAL80^ts^/+ / egg = 30A-GAL4>UAS-RFP, G-trace/+; GAL80^ts^/UAS-egg RNAi / Nurf = 30A-GAL4>UAS-RFP, G-trace/+; GAL80^ts^/UAS-Nurf-38 RNAi#1 / Nurf#2 = 30A-GAL4>UAS-RFP, G-trace/+; GAL80^ts^/UAS-Nurf-38 RNAi#2 / Nurf; STAT/+ = 30A-GAL4>UAS-RFP, G-trace/ GAL80^ts^; UAS-Nurf-38 RNAi#1/ STAT92E^06346^ / Nurf STAT = UAS-STAT RNAi/+; 30A-GAL4>UAS-RFP, G-trace/UAS-Dcr; GAL80^ts^/ UAS-Nurf RNAi#1. / Axin = 30A-GAL4>UAS-RFP, G-trace/+; GAL80^ts^/UAS-Axin. (D) The discs were processed identically, to allow comparison of GFP intensity. GAL4 only = STAT-GFP/30A-GAL4; GAL80^ts^/+ (no G-trace). Nurf-38 = STAT-GFP/30A-GAL4; GAL80^ts^/UAS-Nurf-38 RNAi#1 (no G-trace). Brackets indicate the notum and the arrows, the pouch. Scale bar = 50 μm. (E) The extent of translocation of hinge cells into the pouch was quantified by measuring the area of GFP^+^RFP^-^ cells in the pouch and normalizing it to the total GFP^+^RFP^+^ area of the hinge for each disc. Please see S3 Fig for details on quantification. Student t-test was used to compute p values. (F) GFP intensity in the notum, the dorsal hinge, or the pouch were quantified in images such as those in D and averaged from 15 discs per treatment/genotype in three different experiments (biological replicates). p values shown are against–IR samples of the same genotype. Please see S3 Fig for details on quantification. The difference between GAL4+IR and Nurf+IR was not statistically significant (p = 0.22–0.29 for notum, hinge and pouch).

Our previous work found that Wg and STAT protected the hinge from IR-induced apoptosis and promoted the translocation of hinge cells into the pouch [14]. Therefore, we investigated whether Wg and STAT also has a role in the generation of ectopic wing discs after irradiation. Depletion of STAT with RNAi in the 30A domain reduced the frequency of classes II-IV (Fig 5A, compare the first two bars). We conclude that STAT activity is required in the RFP^+^ cells to produce ectopic wings. We also inhibited Wg by expressing the antagonist Axin using the protocol in Fig 1I. We found, however, that the resulting discs were malformed even without irradiation. We reasoned that Axin, being an inhibitor, would not require the lag time RNAi needs to take effect in other experiments. As such, Wg, we believe, was being inhibited too early in larval development. In contrast, STAT RNAi produced wing discs that were indistinguishable from GAL4 only controls in un-irradiated samples (S2 Fig). Therefore, we reared larvae for an additional 24 h before shifting to 29° in UAS-Axin experiments. This protocol produced discs that were indistinguishable from GAL4 only controls or STAT RNAi discs (S2 Fig). Under these conditions, UAS-Axin reduced the frequency of ectopic discs compared to similarly treated GAL4 only controls (Fig 5B).

### Epigenetic regulators make cell-autonomous contributions to ectopic growth

The generation of ectopic wing discs thus described involves changes in cell fate, gene expression status (RFP^+^ to RFP^-^) or both. Consistent, we found that depletion of epigenetic regulators of transcription altered the frequency of ectopic wings (Fig 5A). We depleted Enhancer of Polycomb, E(Pc); eggless (egg) and Set2, encoding histone methyl transferases; or Nurf-38, encoding a member of the Nucleosome Remodeling Factor (NURF) complex. These regulators were chosen because prior studies showed them to have a role in the apoptosis of larval salivary gland [24], and we wanted to know if these also affect IR-induced apoptosis in the wing discs. Depletion of each using published RNAi constructs produced inconclusive effects on apoptosis. Instead we saw a significant increase in the frequency of ectopic discs at 72 h after irradiation upon depletion of egg, Set2 or Nurf-38, but not E(Pc), compared to GAL4 only controls (Fig 5A). No ectopic discs were produced without IR (Fig 5C). Of these, Nurf-38 had the strongest effect and was confirmed using a second RNAi line (‘Nurf#2’). Therefore, subsequent analyses focused on Nurf-38.

### Normal and abnormal regeneration are genetically separable

Two genes that showed the strongest effect on the frequency of ectopic growths are STAT and Nurf-38. Further, they act in an opposing manner; depletion of STAT inhibited ectopic growths while depletion of Nurf-38 promoted them. To ask if these are functionally related, we depleted both simultaneously. Reduction of STAT using heterozygotes for a STAT severe loss-of-function allele [25, 26] rescued the incidence of ectopic growth caused by 30A>Nurf-38 RNAi (Fig 5A, the second to last bar). This interaction was confirmed with simultaneous RNAi for STAT and Nurf-38 (Fig 5A, the last bar). These results are consistent with STAT functioning downstream or parallel to Nurf-38 to produce ectopic growths, with each providing activities that oppose the other.

Our published studies found that STAT was needed for hinge cells to translocate to the pouch during normal regeneration [14]. This is seen by quantifying the extent of translocation of RFP^-^GFP^+^ hinge cells into the pouch as in Fig 1D (quantification method described in S3 Fig). Under conditions where depletion of Nurf-38 led to increased ectopic discs, there was no significant effect on the movement of hinge cells into the pouch (Fig 5E, compare GAL4 only to Nurf), suggesting that depletion of Nurf-38 was unable to promote regeneration beyond what was normally seen. Thus, normal and abnormal regeneration show different dependence on Nurf-38. We next asked if Nurf-38 depletion could promote regeneration under conditions where regeneration was defective. To this end, we analyzed discs in which Nurf-38 and STAT were depleted simultaneously in the 30A domain. Such discs show reduced ectopic growth suggesting that STAT was successfully depleted (Fig 5A, the last bar). In the same discs, translocation of hinge cells into the pouch was significantly greater than in discs with STAT RNAi alone (Fig 5E, compare STAT to Nurf STAT). We conclude that depletion of Nurf-38 could indeed rescue regeneration defects that result from depletion of STAT.

Genetic interaction data described above suggest that STAT functions parallel or downstream of Nurf-38 in ectopic disc formation. To distinguish between these possibilities, we assayed the effect of Nurf-38 depletion on STAT. STAT activity could be monitored using a GFP transcriptional reporter with 10X STAT binding sites from a known STAT target gene, Socs36E (‘STAT-GFP’, [27]). This reporter is highly expressed in the hinge as reported previously (Fig 5D, GAL4 only -IR). Irradiation increased STAT-GFP reporter expression throughout the disc, but more obviously in the notum (brackets) and the pouch (arrows) that normally display close to background levels (Fig 5D, quantified in F; see S3 Fig for quantification method). In discs expressing 30A-GAL4>Nurf-38 RNAi, STAT-GFP also increased after irradiation and this change was similar to what was seen in GAL4 only controls (Fig 5D, quantified in F). We conclude that while depletion of Nurf-38 could modulate a STAT-dependent process in the growth of ectopic discs, this effect is not through changes in STAT activity as detected by the Socs36E-GFP reporter. In other words, these data favor the possibility that STAT and Nurf-38 function in parallel in ectopic disc formation.

### Ectopic growths show induction of hinge marker Zfh2

**Fig 6:**
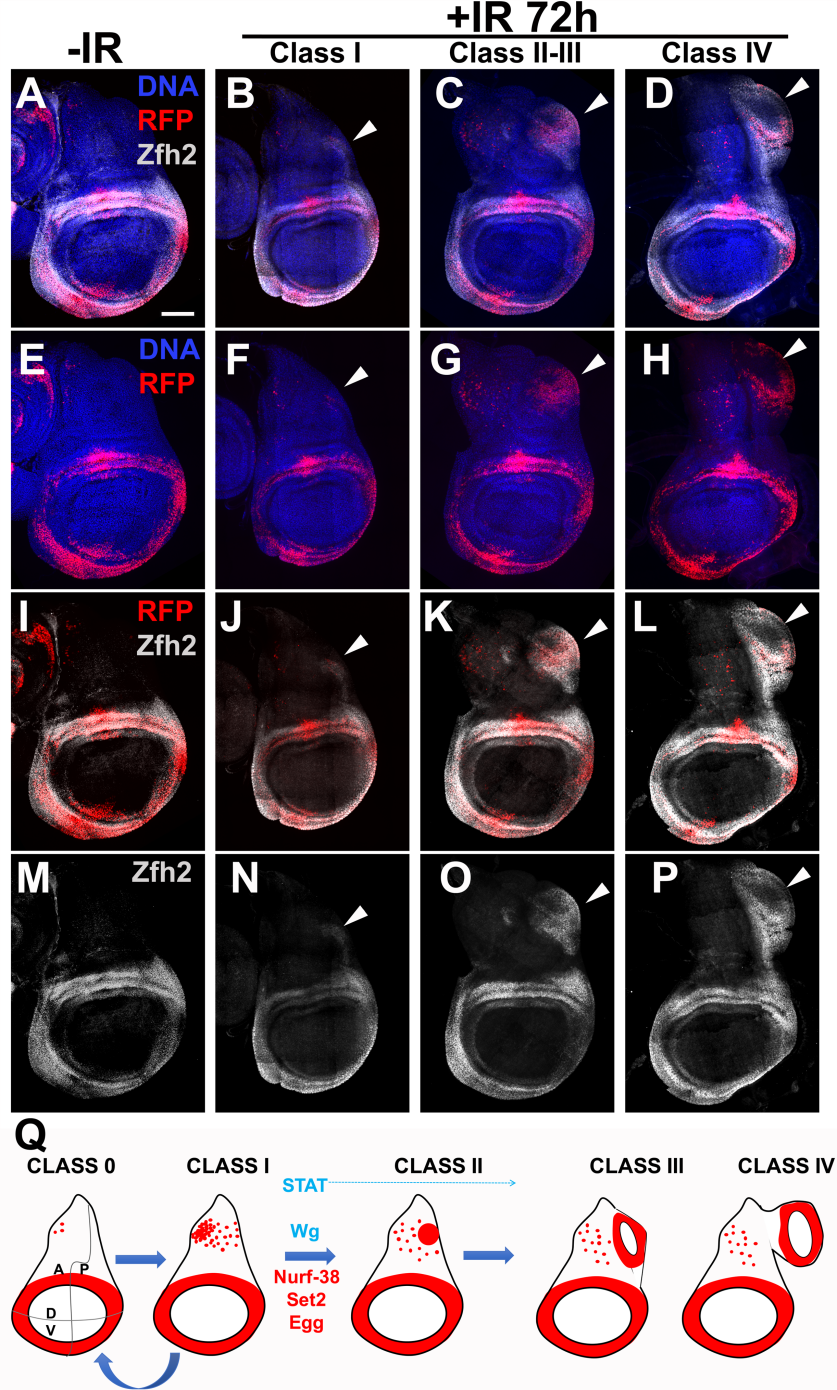
The presence of Zfh2 on ectopic growths and a model for ectopic disc formation. (A-P) Zfh2 staining in primary and ectopic discs. Larvae of the genotype 30A-GAL4>UAS-RFP, G-trace/+; GAL80^ts^/+ were treated as in Fig 1I. The discs were dissected 72 h after irradiation, fixed and stained for DNA and with an antibody against Zfh2. The discs were also visualized for RFP. Zfh2 signal is absent in the notum as expected but appears in ectopic growths (arrowheads). Scale bar = 50 μm. (Q) A model for the formation of ectopic wing discs after irradiation. STAT and Wg promote the process (blue) while Nurf-38, Set2 and Egg inhibit it (red).

To ask whether 30A-GAL4 expression in the notum and later in the ectopic discs indicates hinge fate, we stained for the hinge marker Zfh2. Zfh2 is a transcription factor and a downstream effector of STAT during wing development [28] and during regeneration of genetically ablated wing pouch [9]. Zfh2 expression is confined to the hinge in the 3^rd^ instar wing disc where gray Zfh2 signal encompasses and extends beyond the red 30A>RFP domain (compare Fig 6E and 6M). Without irradiation, we saw little or no signal in the notum despite the presence of RFP^+^ cells in the region (Fig 6, -IR panels). In irradiated discs, Zfh2 was detectable in the notum. Specifically, RFP^+^ cells in the posterior half of the notum in class I discs show faint Zfh2 signal, which also encompassed the RFP signal and extended to neighboring cells (arrowhead in Fig 6B and corresponding panels below). In class II/III discs where the posterior RFP^+^ cell cluster was clearly visible, Zfh2 also encompassed and extended beyond the RFP signal (arrowhead in Fig 6C and corresponding panels below). The RFP^+^ ectopic hinge in class IV discs likewise showed Zfh2 staining (arrowhead in Fig 6D and corresponding panels below). Based on the presence of Zfh2, we conclude that 30A>RFP-expressing cells in the ectopic discs did indeed take on the hinge fate.

These data lead us to propose a model for ectopic disc growth (Fig 6Q). In this model, irradiation increases RFP^+^ cells in the notum through increased mitosis such that all discs are class I by 24 h after irradiation (Fig 3A). Class I discs could remain as class I, revert to class 0, or form a posterior cell cluster to progress to class II. Cells of the cluster then produce an ectopic hinge/pouch to progress to classes III/IV. We propose that the formation of the cluster and subsequent maturation of it into an ectopic disc requires STAT; depletion of STAT not only reduced the fraction of class II-IV discs compared to GAL4 only controls but also abolished classes III and IV. Therefore, STAT likely functions in all step starting with the formation of class II. Inhibition of Wg also reduced the overall fraction of Class II-IV (like STAT RNAi), but still was compatible with the formation of classes III/IV (unlike STAT RNAi). This suggests that Wg activity is needed in the 30A domain for the formation of the cluster, but less so for subsequent steps. We cannot rule out, however, that Wg activity is needed in cells outside of the 30A domain for the cluster of RFP^+^ cells to mature into an ectopic wing disc. We further propose that Set2, Nurf-38 and Egg oppose the formation of class II discs as well as subsequent steps; thus, depletion of Set2, Nurf-38 or Egg increased classes II-IV.

## Discussion

Regeneration inevitably requires cell fate changes. During blastema formation in vertebrate limb development, for example, cells of the stump de-differentiate, migrate to the wound site, proliferate, and re-differentiate to new fates [29]. Cell fate changes would require destabilizing the gene expression landscape of differentiated cells and, later, re-establishing and stabilizing a new gene expression landscape. In different experimental systems including *Drosophila* and vertebrates, post-translational modifications in Histones correlate with cell fate changes during tissue regeneration (e.g. [30]). Where studied, there are also causal relationships, for example the role of Polycomb/Trithorax group of genes in transdetermination and regeneration (e.g. [31–36]). In this context, our results contribute to our understanding of regeneration after damage by ionizing radiation as follows.

We report a remarkable feat of cellular gymnastics that occur during a 48 h period, from 24 to 72 h after irradiation, to produce an ectopic wing disc. This can happen without the full benefit of cell multiplication (in Rux-expressing discs). While we cannot rule out translocation of hinge cells into the notum in the formation of ectopic discs, our findings do not favor this possibility as described above (Fig 4). Instead, a possible mechanism is the de novo induction of the hinge marker 30A-GAL4 in the notum cells to produce the posterior RFP^+^ cell clump. This, we believe is the critical step that is governed by STAT, Wg, Set2, Egg and Nurf-38 (model in Fig 6Q). Once formed, this cluster of RFP^+^ cells can lose their hinge fate (become RFP^-^GFP^+^) and recruit un-related cells (RFP^-^GFP^-^) to form an ectopic wing disc (Fig 2I). Moreover, the genetic requirements we identified are cell-autonomous; we expressed dsRNA and Axin using the 30A-GAL4 driver that also drove RFP expression. Thus, while STAT activity increases throughout the notum after irradiation (Fig 5D), cell autonomous depletion within RFP^+^ cells was sufficient to interfere with duplication, thereby identifying a cell population critical for the formation of ectopic wing discs.

Are ectopic discs duplications or transdeterminations? Because we see two wing discs instead of one, we may classify ectopic discs as duplications. On the other hand, if duplication means copying of existing structures, ectopic discs are not exactly duplications; ectopic pouch, for example, is not derived from the primary pouch. Likewise, notum to hinge/pouch fate change we see may be classified as transdetermination. On the other hand, if transdetermination is defined as when one disc takes on the fate of another (e.g. leg->wing), ectopic discs that remain as wing disc do not fit this definition. Therefore, we will refer to what we see simply as ‘ectopic discs’, to avoid confusion.

The frequency of ectopic wing discs after irradiation (~20% in both GAL4 only and *w*^*1118*^) seems high but is in fact lower than the frequency of abnormal regenerations recorded in classical studies of surgically ablated discs. Schubiger saw, for example, that among lateral (posterior) leg fragments cultured in the adult abdomen, 33/41 (80%) regenerated discs with duplications [3]. In the same study, among medial (anterior) leg fragments cultured in the adult abdomen, 21/42 (50%) regenerated discs with transdetermination. Classical regeneration studies using X-rays to cause damage also describe disc duplications but these observations were limited to irradiation of ‘early imaginal discs consisting of only about 20 cells’ [13].

We describe a step-wise sequence of events that can lead to mistakes during regeneration, i.e. ectopic discs. A key step in this sequence, which we propose is targeted by epigenetic regulators, STAT and Wg, is the formation of the posterior cell cluster. Wg, STAT and their homologs have conserved well-studied roles during normal development as well as in tissue regeneration. Wg and STAT are also implicated in pattern duplication and transdetermination of larval imaginal discs. In fact, ubiquitous expression of Wg in second and third instar larva is sufficient to induce transdetermination in imaginal discs [37, 38]. Molecular analysis of regeneration after surgical ablation of leg discs also identified prominent roles for both STAT and Wg [8]. Here, JNK activation at the wound site activates JAK/STAT and Wg, both of which then contribute to extra proliferation to form a blastema. We also identified STAT and Wg is essential for the hinge cells to participate in regeneration of the pouch after IR damage [14]. The current study further solidified the role of STAT and Wg in regeneration, by identifying their contribution to abnormal regenerations induced by IR.

All ectopic wing discs we saw grew out of the dorsal posterior notum. Their location and appearance resemble ‘supernumerary wings discs’ that result from ectopic expression of Wg [39, 40] or STAT [41], or mutations in *Drosophila* EGFR (DER) [42]. In the first case, expression of UAS-Wg, with Dpp-GAL-4 driver or in Ubx-driven clones, produced a wing disc that grows out of the notum. In the second study, expression of Dpp-GAL4>UAS-Upd, a ligand for JAK/STAT, or en-GAL4>UAS-hop (Dm JAK), also led to the growth of ectopic wings out of the notum. The key difference was that in the published studies re-programming of cells in the notum to wing pouch was limited to a very early stage in wing development. For example, Wg overexpression at 36±6 h after egg laying induced ectopic wing discs but could not at 48±6 h. The authors concluded that Wg has an early role in specifying wing pouch at the expense of the notum but that at later times, Wg has another role, in D/V patterning. Likewise, induction of ectopic wings was achieved by expression of Upd in second instar larvae. Likewise, ectopic wing discs result from the loss of DER only in young larvae (up to 120 h after egg laying at 17°C or 72 h after egg laying under ‘normal culture conditions’) [42]. In our studies, larvae at the time of irradiation were 72–96 h old. Taken together, these results suggest that in irradiated discs, Wg and STAT revert to their potential seen earlier in development.

NURF is known to oppose JAK/STAT signaling in the innate immune response in Drosophila, specifically in the larval hemocytes and the fat body [43]. In those tissues, NURF is recruited to a subset of STAT target genes by physical association with Zinc-finger protein Ken, and provides a repressive function. JAK/STAT signaling is activated in response to infection in order to mount an effective immune response. NURF is proposed to temper JAK/STAT such that an immune response occurs only when needed and is shut off when no longer necessary. This, we propose, parallels the activation of STAT after irradiation that serves an essential regenerative function but must be tempered in order to prevent excessive regeneration, i.e. the production of ectopic structures. Nurf-38, we found, also opposes STAT in this context, in genetics and RNAi analyses. Depletion of Nurf-38, however, did not affect bulk STAT activity as seen with the STAT-GFP reporter derived from one STAT target gene. It remains possible that Nurf-38 instead affects STAT activity in the context of another STAT target gene (s), which is important for regeneration. While our data implicate epigenetic regulators in abnormal regeneration, we do not yet know whether epigenetic regulation is at play. Identification of STAT and Nurf-38 targets and analysis of their epigenetic status would be required to address this possibility.

We note that we do not believe regeneration in the wing disc occurs by polyploidization. Rather, polyploidization in our experiments was simply an outcome of using Rux to block mitosis. We were surprised by the result that mitosis was dispensable for the formation of an ectopic wing disc. But there is precedent for regeneration with polyploid cells [44]. For example, wound closure in *Drosophila* adult abdomen occurs partly through polyploidization and cell fusion [45]. Ectopic discs in UAS-Rux experiments in our experiments served a useful purpose in that these provide strong evidence that ectopic pouches were made of both cells from the 30A-GAL4 domain (large, RFP^+^) and recruits from outside (small, GFP^-^RFP^-^, arrow in Fig 4G). In this regard, the RFP^+^ posterior cluster of cells may resemble the Spemann-Mangold Organizer, identified by early Experimental Embryologists as a region of a frog embryo that, when surgically implanted at an ectopic location, could induce a second body axis. The resulting supernumerary organs included not only the cellular descendants of the Organizer but also host cells near the implant site that assumed new fates and were organized into new structures. Loss or gain-of-function studies that test the effect of losing the RFP^+^ cell cluster or its appearance at ectopic locations would be needed to further address this parallel.

## Materials and methods

### Drosophila stocks and methods

These stocks are described in Flybase: *w*^*1118*^, 30A- GAL4 (on Ch II, Bloomington stock# or BL37534), Ptub-GAL80^ts^ on Ch III, 10XSTAT-GFP (on Ch II, [27]), UAS-Axin-GFP (on Ch III, BL7225), UAS-STAT RNAi (on X, BL26899), Set2 RNAi (BL55221), egg RNAi (BL31352), Nurf-38 RNAi (#1 = BL35444; #2 = BL31341), E(Pc) RNAi (BL28686); UAS-Rux (BL9166); *STAT92E*^*06346*^ [25, 26]. All Nurf-38 RNAi experiments used BL35444 with the exception of Fig 5A ‘Nurf-38 #2’. The stock used for lineage tracing is also described in Flybase; w*; P{UAS-RedStinger}4, P{UAS-FLP.D}JD1, P{Ubi-p63E(FRT.STOP)Stinger}9F6 /CyO (BL28280). Genotypes for BL stocks are in S2 Table.

30A-GAL4>UAS-RFP, G-trace/CyO-GFP; GAL80^ts^/GAL80^ts^ virgin females were crossed to *w*^*1118*^ males (GAL4 only controls) or UAS-dsRNA males. STAT RNAi virgin females were crossed to 30A-GAL4>UAS-RFP, G-trace/CyO-GFP; GAL80^ts^/GAL80^ts^ males. Progeny bearing G-trace (RFP^+^GFP^+^ larvae) were sorted for use.

### Larvae culture and irradiation

Larvae were raised on Nutri-Fly Bloomington Formula food (Genesee Scientific) at 25°C unless otherwise noted. The cultures were monitored daily for signs of crowding, typically seen as ‘dimples’ in the food surface as larvae try to increase the surface area for access to air. Cultures were split at the first sign of crowding.

Larvae in food were irradiated in a Faxitron Cabinet X-ray System Model RX-650 (Lincolnshire, IL) at 115 kv and 5.33 rad/sec.

### Antibody staining

Antibodies to cleaved Phospho-S10-Histone H3 (1:1000, rabbit monoclonal, Upstate Biotech), Wingless (1:100, mouse monoclonal, *Drosophila* Hybridoma Bank Cat#4D4), Ubx (1:750, Developmental Hybridoma Bank Cat#UbxFB3.38-c), and Zfh2 (1:400, rat polyclonal, [46]). Secondary antibodies (Jackson) were used at 1:100 (rabbit and mouse) or 1:200 (rat). For antibody staining, wing discs were dissected in PBS, fixed in 4% para-formaldehyde in PBS for 30 min, and washed three times in PBS, permeabilized in PBTx (0.5% Triton X-100) for 10 min and rinsed in PBTx (0.1% Triton X-100). The discs were blocked in 5% Normal Goal Serum in PBTx (0.1% Triton X-100) for at least 30 min and incubated overnight at 4°C in primary antibody in block. The discs were rinsed thrice in PBTx (0.1% Triton X-100) and incubated in secondary antibody in block for 2 h at room temperature. Stained discs were washed in PBT. The discs were counter-stained with 10 μg/ml Hoechst33258 in PBT or PBTx (0.1%TritonX-100) for 2 min, washed 3 times, and mounted on glass slides in Fluoromount G (SouthernBiotech). Discs that were imaged without antibody staining (in quantification of disc classes) were fixed in 10% paraformaldehyde in PBS for 10 min, washed 1xPBS for 10 min and washed in PBTx for 5 min. The discs were stained with Hoechst 33258 and mounted as described above.

### Image analysis

With the exceptions noted below, the discs were imaged on a Perkin Elmers spinning disc confocal attached to a Nikon inverted microscope, using a SDC Andor iXon Ultra (DU-897) EM CCD camera. The NIS- Elements acquisition software’s large image stitching tool was used for the image capture. 20–21 z-sections 1 um apart were collected per disc and collapsed using ‘maximum projection’ in Image J. The exceptions are Fig 3, Fig 4E–4H and 4M–4O that show a single z-section each; Fig 1A, 1B, 1E, 1F and 1H and Fig 2D–2F which were acquired on a Leica DMR compound microscope using a Q-Imaging R6 CCD camera and Ocular software.

### Statistical analysis

The distribution of disc classes was analyzed using the Chi-square test, using the numbers for GAL4 only control +IR to generate the expected ratios of classes. Cell number in the notum, RFP^+^ area, and STAT-GFP were analyzed using a 2-tailed t-test. For sample size justifications, we used a simplified resource equation from [47]; E = Total number of animals − Total number of groups, where E value of 10–20 is considered adequate. When we compare two groups (-/+IR or GAL4 vs RNAi, for example), 6 per group or E = 11 would be adequate. All samples subjected to statistical analysis meet or exceed this criterion.

## Supporting information

S1 Fig Rux blocks mitosis. **Related to Fig 3.** Larvae were treated as shown in Fig 1I and dissected 24 h after a shift to 29°C. The discs were fixed and stained for DNA and visualized for RFP. Genotype: 30A-GAL4>UAS-RFP, G-trace/+; GAL80^ts^/+ (A-C) and 30A-GAL4>UAS-RFP, G-trace/UAS-Rux; GAL80^ts^/+ (D-F). Disc margins were traced from DNA images. Boxed sections in C and F are magnified 3X, black/white inverted for ease of viewing, and shown in C’ and F’ respectively. All images are Dorsal up and Posterior to the right of the viewer. Scale bar = 120 μm in A-F and 40 μm in C’ and F’.
(PDF)

S2 Fig The temperature shift protocol is compatible with disc development. **Related to Fig 5.** (A-B) Larvae were treated as shown in Fig 1I and dissected 72 h after irradiation with 0 R (un-irradiated controls). The discs were fixed and stained for DNA and visualized also for RFP. Genotype: 30A-GAL4>UAS-RFP, G-trace/+; GAL80^ts^/+ in (A) and UAS-STAT RNAi/+; 30A-GAL4>UAS-RFP, G-trace/+; GAL80^ts^/+ in (B). (C) Larvae were treated as in Fig 1I except for one modification: larvae were aged for 72 h at 25°C from the end of egg collection rather than from the beginning of egg collection. Thus, the larvae were 72–80 h old at the time of temperature shift to 29°C. Genotype: 30A-GAL4>UAS-RFP, G-trace/+; GAL80^ts^/ UAS-Axin. Scale bar = 50 μm.
(PDF)

S3 Fig Quantification of translocation and STAT-GFP. **Related to Fig 5.** (A-B) Wing discs from–IR (A) and +IR (B) larvae are shown to illustrate how translocation of hinge cells into the pouch (arrow) was quantified. The area of GFP^+^RFP^-^ cells within the circle was quantified in Image J and divided by the area of RFP^+^GFP^+^ cells in the hinge. (C) A wing disc showing STAT-GFP reporter expression. Average fluorescence in the circle was quantified in Image J from the notum (1), the hinge (2), and the pouch (3). Background fluorescence was quantified from three locations (4–6), averaged and subtracted from (1–3).
(PDF)

S1 Table Chi-square values. **Related to Figs 1 and 5.** The frequency of disc classes observed after genotype/treatment (Column 1) was tested for significant differences from the expected (Column 2) using a Chi-square test. For values in column 3, five disc classes were taken separately. For values in column 4, the discs were binned into either IR-induced (class II-IV) or others (classes 0/I). p values that correspond to each chi-square value are provided as: ns = not significant, *p<0.05, **p<0.01, ***p<0.001. Considering the five classes separately, we believe, can provide false positives because the differences in the frequencies of non-IR-dependent classes, 0 or I, could contribute to the chi square value. Therefore, more conservative values in column 4 (bold) were used in the main text.
(PDF)

S2 Table Stocks from Bloomington Stock Center used in this work. (PDF)
