# Supplementary figures and images for: STAT, Wingless, and Nurf-38 determine the accuracy of regeneration after radiation damage in Drosophila
Source: PLoS Genet. 2017 Oct 13;13(10):e1007055. doi: 10.1371/journal.pgen.1007055 (PMC5656321; doi:10.1371/journal.pgen.1007055)

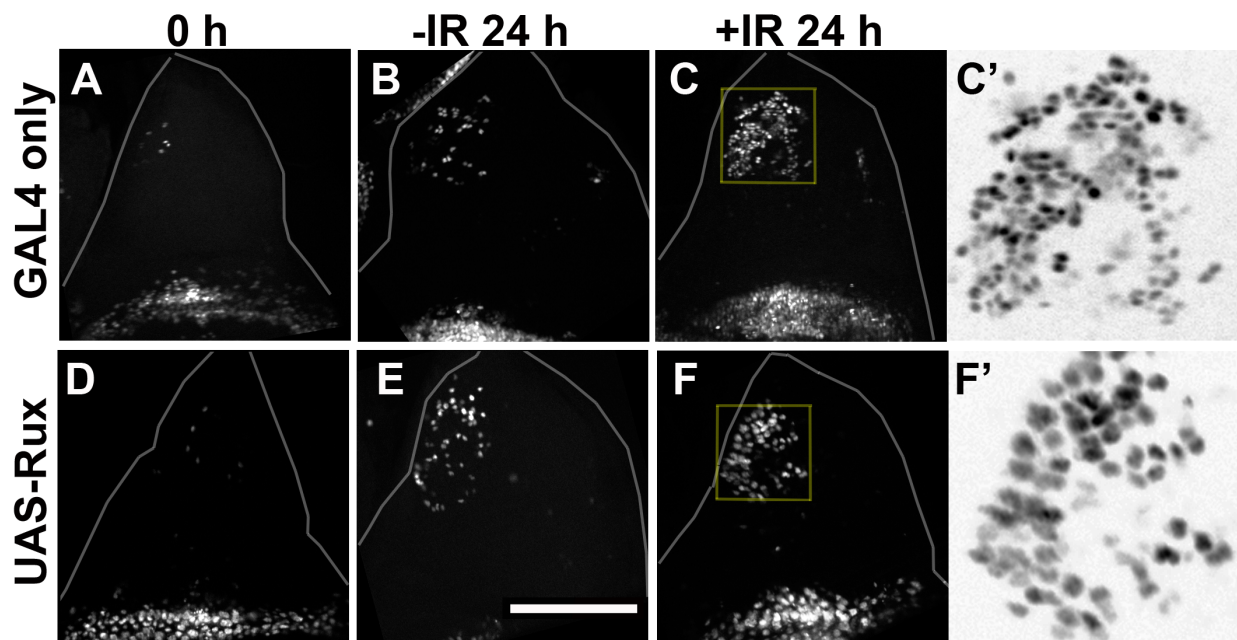

**S1 Fig. Rux blocks mitosis. Related to Fig 3.**

Supplement: S1 Fig — Related to Fig 3. Larvae were treated as shown in Fig 1I and dissected 24 h after a shift to 29°C. The discs were fixed and stained for DNA and visualized for RFP. Genotype: 30A-GAL4>UAS-RFP, G-trace/+; GAL80ts/+ (A-C) and 30A-GAL4>UAS-RFP, G-trace/UAS-Rux; GAL80ts/+ (D-F). Disc margins were traced from DNA images. Boxed sections in C and F are magnified 3X, black/white inverted for ease of viewing, and shown in C’ and F’ respectively. All images are Dorsal up and Posterior to the right of the viewer. Scale bar = 120 μm in A-F and 40 μm in C’ and F’. (PDF) [file pgen.1007055.s001.pdf]

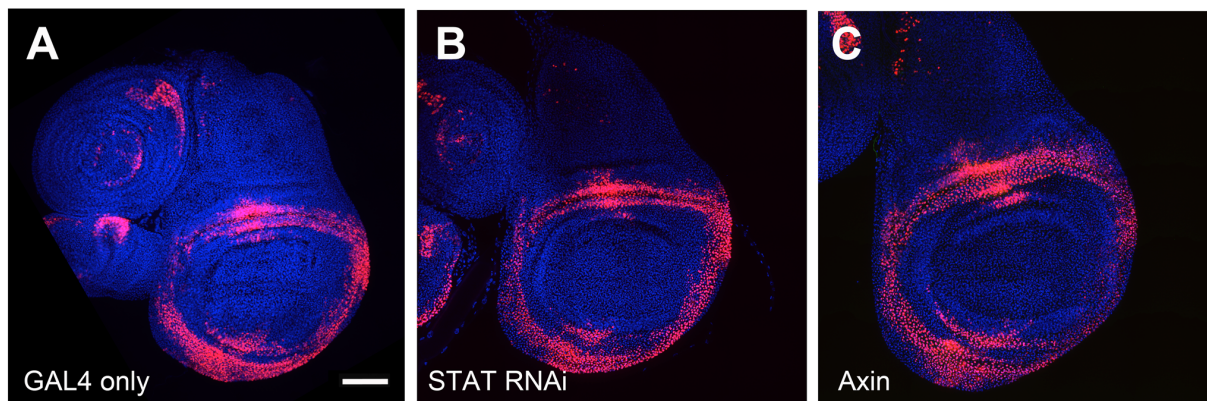

**S2 Fig. The temperature shift protocol is compatible with disc development. Related to Fig 5.**

Supplement: S2 Fig — Related to Fig 5. (A-B) Larvae were treated as shown in Fig 1I and dissected 72 h after irradiation with 0 R (un-irradiated controls). The discs were fixed and stained for DNA and visualized also for RFP. Genotype: 30A-GAL4>UAS-RFP, G-trace/+; GAL80ts/+ in (A) and UAS-STAT RNAi/+; 30A-GAL4>UAS-RFP, G-trace/+; GAL80ts/+ in (B). (C) Larvae were treated as in Fig 1I except for one modification: larvae were aged for 72 h at 25°C from the end of egg collection rather than from the beginning of egg collection. Thus, the larvae were 72–80 h old at the time of temperature shift to 29°C. Genotype: 30A-GAL4>UAS-RFP, G-trace/+; GAL80ts/ UAS-Axin. Scale bar = 50 μm. (PDF) [file pgen.1007055.s002.pdf]

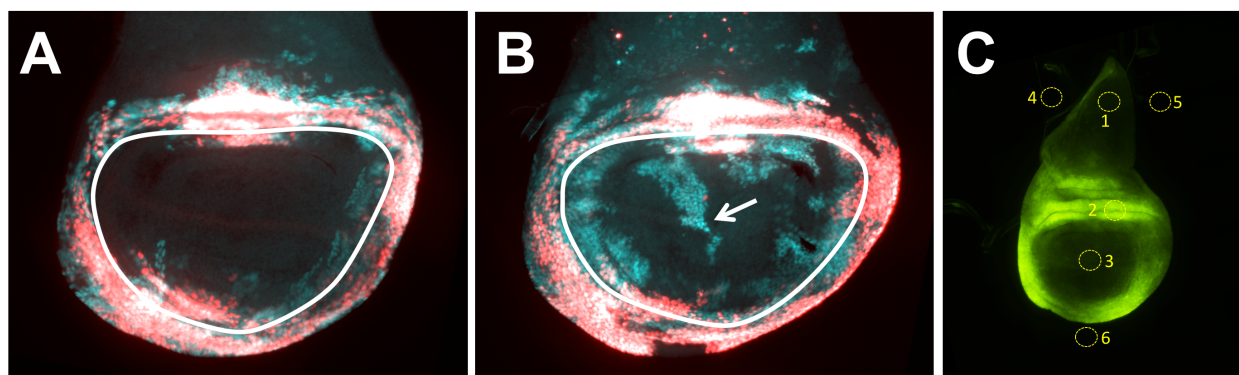

**S3 Fig. Quantification of translocation and STAT-GFP. Related to Fig 5.**

Supplement: S3 Fig — Related to Fig 5. (A-B) Wing discs from–IR (A) and +IR (B) larvae are shown to illustrate how translocation of hinge cells into the pouch (arrow) was quantified. The area of GFP+RFP- cells within the circle was quantified in Image J and divided by the area of RFP+GFP+ cells in the hinge. (C) A wing disc showing STAT-GFP reporter expression. Average fluorescence in the circle was quantified in Image J from the notum (1), the hinge (2), and the pouch (3). Background fluorescence was quantified from three locations (4–6), averaged and subtracted from (1–3). (PDF) [file pgen.1007055.s003.pdf]
