# Supplementary material for: STAT, Wingless, and Nurf-38 determine the accuracy of regeneration after radiation damage in Drosophila
Source: PLoS Genet. 2017 Oct 13;13(10):e1007055. doi: 10.1371/journal.pgen.1007055 (PMC5656321; doi:10.1371/journal.pgen.1007055)
Supplement: S1 Table — Related to Figs 1 and 5. The frequency of disc classes observed after genotype/treatment (Column 1) was tested for significant differences from the expected (Column 2) using a Chi-square test. For values in column 3, five disc classes were taken separately. For values in column 4, the discs were binned into either IR-induced (class II-IV) or others (classes 0/I). p values that correspond to each chi-square value are provided as: ns = not significant, *p<0.05, **p<0.01, ***p<0.001. Considering the five classes separately, we believe, can provide false positives because the differences in the frequencies of non-IR-dependent classes, 0 or I, could contribute to the chi square value. Therefore, more conservative values in column 4 (bold) were used in the main text. (PDF) [file pgen.1007055.s004.pdf]

| Column 1 | Column 2 | Column 3 | Column 4 |
|---|---|---|---|
| Genotype/Treatment (observed) | p value against (expected) | Class 0-IV separately (Df=4) | **IR-induced classes II-IV together (Df=1)** |
| E(Pc) RNAi +IR | GAL4>G-trace +IR | 29.800*** | **1.0238 (ns)** |
| Set2 RNAi +IR | GAL4>G-trace +IR | 31.850*** | **13.390*** ** |
| Egg RNAi +IR | GAL4>G-trace +IR | 34.490*** | **16.307*** ** |
| Nurf-38 RNAi +IR | GAL4>G-trace +IR | 86.846*** | **44.261*** ** |
| Nurf-38 RNAi #2 +IR | GAL4>G-trace +IR | 61.746*** | **19.961*** ** |
| STAT RNAi +IR | GAL4>G-trace +IR | 29.180*** | **11.614*** ** |
| Axin +IR | GAL4>G-trace +IR | 22.469*** | **17.566*** ** |
| STAT RNAi +IR | Nurf-38 RNAi; STAT/+ +IR | 9.189 (ns) | **1.028 (ns)** |
| STAT RNAi +IR | Nurf-38 STAT RNAi +IR | 200.255*** | **4.130 (ns)** |
| Nurf-38 STAT RNAi +IR | Nurf-38 RNAi | 160.732*** | **61.191*** ** |

**S1 Table. Chi-square values. Related to Fig 1 and 5.**
