# Supplementary material for: STAT, Wingless, and Nurf-38 determine the accuracy of regeneration after radiation damage in Drosophila
Source: PLoS Genet. 2017 Oct 13;13(10):e1007055. doi: 10.1371/journal.pgen.1007055 (PMC5656321; doi:10.1371/journal.pgen.1007055)
Supplement: S2 Table — (PDF) [file pgen.1007055.s005.pdf]

Bloomington stock number	Figures	Genotype
7225	Fig. 5	w[*]; Kr[If-1]/CyO; P{w[+mC]=UAS-Axin.GFP}3/TM3, Sb[1]
26899	Fig. 5	P{w[+mC]=UAS-Stat92E.RNAi}1, w[*]
55221	Fig. 5	y[1] v[1]; P{y[+t7.7] v[+t1.8]=TRiP.GLC01838}attP40
31352	Fig. 5	y[1] v[1]; P{y[+t7.7] v[+t1.8]=TRiP.JF01310}attP2
35444	Fig. 5	y[1] sc[*] v[1]; P{y[+t7.7] v[+t1.8]=TRiP.GL00369}attP2
31341	Fig. 5	y[1] v[1]; P{y[+t7.7] v[+t1.8]=TRiP.JF01299}attP2
28686	Fig. 5	y[1] v[1]; P{y[+t7.7] v[+t1.8]=TRiP.JF03101}attP2
9166	Fig. 3, 4	w[*]; P{w[+mC]=UAS-rux.T}C1/SM6a
11681	Fig. 5	ry[506] P{ry[+t7.2]=PZ}Stat92E[06346]/TM3, ry[RK] Sb[1] Ser[1]
26197	Fig. 5	w[1118]; P{w[+mC]=10XStat92E-GFP}1
37534	Fig. 1-6, Fig. S1-S3	w[*]; P{w[+mW.hs]=GawB}30A/CyO
28280	Fig. 1-6, Fig. S1-S3	[*]; P{w[+mC]=UAS-RedStinger}4, P{w[+mC]=UAS-FLP.D}JD1, P{w[+mC]=Ubi-p63E(FRT.STOP)Stinger}9F6/CyO

S2 Table. Stocks from Bloomington Stock Center used in this work.
